# Supplementary material for: Coupling of Temporal-Check-All-That-Apply and Nose-Space Analysis to Investigate the In Vivo Flavor Perception of Extra Virgin Olive Oil and Carriers’ Impact
Source: Foods. 2025 Jul 1;14(13):2343. doi: 10.3390/foods14132343 (PMC12248991; doi:10.3390/foods14132343)
Supplement: Supplementary file 1 [file foods-14-02343-s001.zip › Figure S3.pdf]

Figure S3. ANOVA mixed. F-value and p-value for factor sample (6 levels) (A) and factor replication (3 levels) (B) of the Time of maximum concentration value from each mass curve

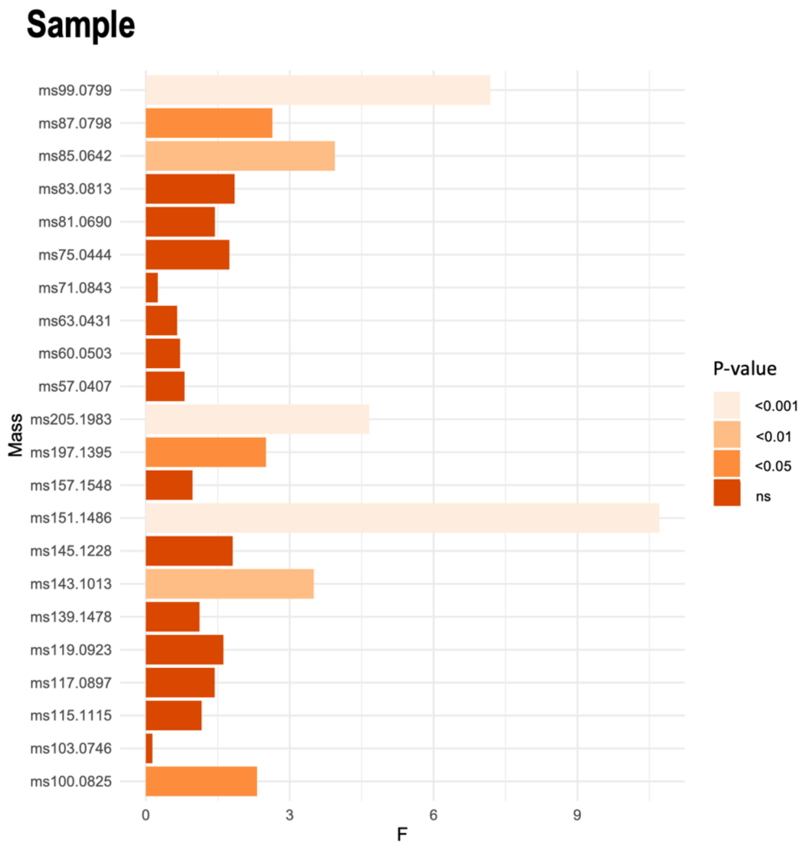

A

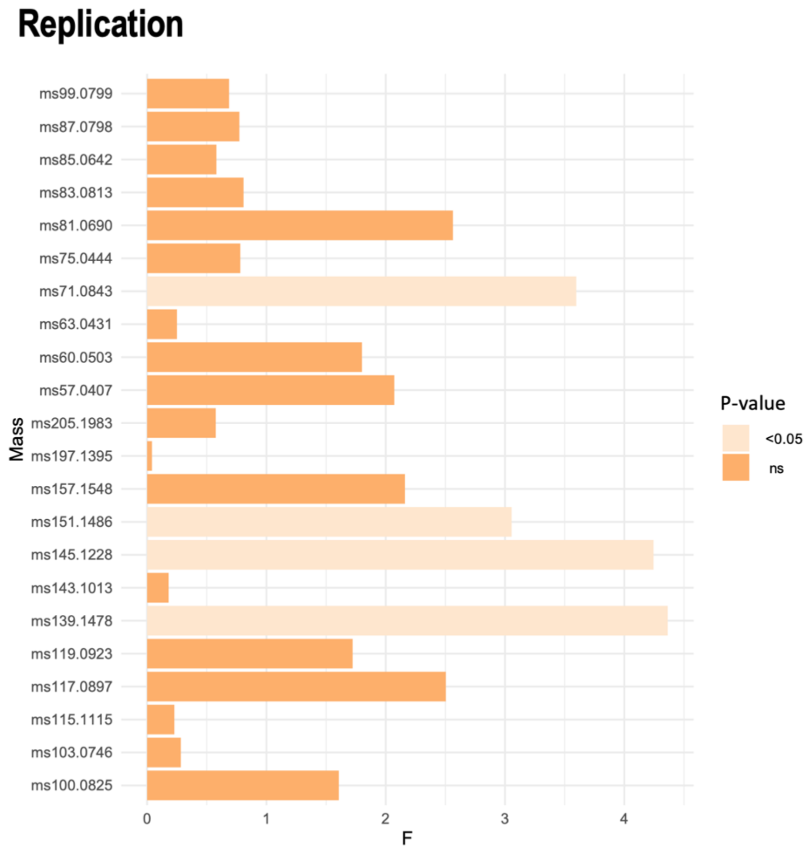

B
